# Statement of Retraction: Prophylactic role of Enhydra fluctuans against arsenic-induced hepatotoxicity via antiapoptotic and antioxidant mechanisms

**DOI:** 10.1080/13510002.2025.2538364

**Published:** 2025-08-04

We, the Editor and Publisher of *Redox Report,* have retracted the following article:

Dua, T. K., Dewanjee, S., & Khanra, R. (2016). Prophylactic role of Enhydra fluctuans against arsenic-induced hepatotoxicity via anti-apoptotic and antioxidant mechanisms. *Redox Report*, 21(4), 147–154. https://doi.org/10.1179/1351000215Y.0000000021

Following publication, concerns were raised by a third party in August 2023 regarding duplication in figure(s) 1C, 2A and 2B. In December 2023, the authors contacted the journal to request a correction.

When the authors were asked for an explanation regarding the above concerns raised, they explained that the images were provided by an outsourced laboratory. In the original published article, there was no declaration of outsourced laboratory work. As a result, the concerns regarding the images remained.

As verifying the validity of published work is core to the integrity of the scholarly record, we are therefore retracting the article. The corresponding author listed in this publication has been informed. The authors do not agree with the retraction.

